# Identification of Phosphorus Stress Related Proteins in the Seedlings of Dongxiang Wild Rice (*Oryza Rufipogon* Griff.) Using Label-Free Quantitative Proteomic Analysis

**DOI:** 10.3390/genes13010108

**Published:** 2022-01-04

**Authors:** Qianwen Deng, Liangfang Dai, Yaling Chen, Decai Wu, Yu Shen, Jiankun Xie, Xiangdong Luo

**Affiliations:** 1 College of Life Science, Jiangxi Normal University, Nanchang 330022, China; dqwsmile@zju.edu.cn (Q.D.); dailf79@163.com (L.D.); yaqing620@163.com (Y.C.); libaibuhecha@163.com (D.W.); sheny202112@163.com (Y.S.); xiejiankun11@163.com (J.X.); 2 College of Agriculture & Biotechnology, Zhejiang University, Hangzhou 310058, China

**Keywords:** Dongxiang wild rice, label-free quantitative proteomic, low phosphorus stress, seedling

## Abstract

Phosphorus (P) deficiency tolerance in rice is a complex character controlled by polygenes. Through proteomics analysis, we could find more low P tolerance related proteins in unique P-deficiency tolerance germplasm Dongxiang wild rice (*Oryza Rufipogon*, DXWR), which will provide the basis for the research of its regulation mechanism. In this study, a proteomic approach as well as joint analysis with transcriptome data were conducted to identify potential unique low P response genes in DXWR during seedlings. The results showed that 3589 significant differential accumulation proteins were identified between the low P and the normal P treated root samples of DXWR. The degree of change was more than 1.5 times, including 60 up-regulated and 15 downregulated proteins, 24 of which also detected expression changes of more than 1.5-fold in the transcriptome data. Through quantitative trait locus (QTLs) matching analysis, seven genes corresponding to the significantly different expression proteins identified in this study were found to be uncharacterized and distributed in the QTLs interval related to low P tolerance, two of which (*LOC_Os12g09620* and *LOC_Os03g40670*) were detected at both transcriptome and proteome levels. Based on the comprehensive analysis, it was found that DXWR could increase the expression of purple acid phosphatases (PAPs), membrane location of P transporters (PTs), rhizosphere area, and alternative splicing, and it could decrease reactive oxygen species (ROS) activity to deal with low P stress. This study would provide some useful insights in cloning the P-deficiency tolerance genes from wild rice, as well as elucidating the molecular mechanism of low P resistance in DXWR.

## 1. Introduction

Phosphorus (P) is one of the essential macronutrients in plant growth and development. It is estimated that 43% (about 5.8 billion hm^2^) of the world’s arable land is deficient in P, and 3/4 farmlands (about 67 million hm^2^) have P shortages in China, which can result in yield reduction by 5–15% (about 25–75 billion kg) [1]. Although soil available P deficiency can be improved by applying phosphate (Pi) fertilizer, the utilization rate of which plants apply it is no more than 20% [2]. This is because most of P in soil exists in the form of insoluble mineral P or bound organic P, which cannot be absorbed by plants. In addition, the main source of Pi fertilizer is Pi rock, which is a non-renewable resource and is expected to be depleted soon, and the heavy use of Pi fertilizer can also cause environmental problems such as eutrophication of water [3].

Plant adaptation to a P-deficiency environment covers a series of gene expression and morphophysiological events [4], such as regulation of P transporters (PTs), mycorrhizal association, phosphatase secretion, organic acid exudation, and alteration in root structure [5]. Studies have shown that OsPHR2 (Phosphate Starvation Response 2), homologous to PHR1 in *Arabidopsis*, is a major transcriptional regulator of low P response in rice [6,7], which could activate the Pi starvation-induced genes including PHT1 (Phosphate Transporter 1) members by binding to the P1BS (PHR1 Binding Sequence; GNATATNC) motif presented in genes’ promoter region [8,9,10,11,12,13].

P-deficiency tolerance, however, is a complex quantitative trait controlled by many genes and is profoundly influenced by the environment [14]. Quantitative trait locus (QTLs) analysis of P-deficiency tolerance related traits in rice showed that there were generated dozens of QTLs in different populations. These QTLs extensively distributed on chromosomes 1, 2, 3, 4, 6, 7, 9, and 12, especially on chromosomes 4, 6, 11, and 12 [15,16,17]. Under different genetic backgrounds, the QTL loci of some related traits overlapped or were adjacent on the same chromosome, indicating that the traits related to low P tolerance had greater heritability.

Based on the results of QTL mapping or fine mapping, Wasaki et al. [18] cloned an *OsPI1* gene on rice chromosome 1; Yi et al. [19] successfully cloned and verified a transcription factor *OsPTF1* that could significantly improve P efficiency in plants; Chin et al. [15] used the molecular marker closely linked to *Pup1* for assisted breeding, and Gamuyao et al. [20] successfully cloned the *PSTOL1* gene. Wissuwa et al. [21] detected 20 P utilization related locus in rice through genome-wide association analysis and identified a candidate gene on chromosome 1 through comparative variation and expression analysis. Meanwhile, some researchers (including our group) constructed interspecific hybrid population with close wild rice and obtained major QTLs for low P tolerance of wild rice, as well as created some new germplasm [22,23], which broadened the genetic diversity of P efficient uptake and utilization in rice and laid an important foundation for the utilization of P efficient genes in wild rice. Therefore, excavating the high efficiency P utilization gene of the crop itself will provide insights in solving the yield problem caused by P deficiency and cultivating new varieties resistant to low P.

Dongxiang wild rice (hereinafter referred to as DXWR) is a common wild rice (*O. rufipogon* Griff.) found in the northernmost distribution latitude to date. It has more abundant genetic diversity than cultivated rice and contains a large number of excellent genes, including low P tolerance genes, some of which do not even exist in cultivated rice [23,24,25,26]. Therefore, DXWR is a valuable resource for the excavation and utilization of low P resistant genes. So far, some QTLs related to low P stress tolerance have been identified in DXWR [23]. In order to understand the molecular mechanism related to low P resistance of DXWR, we detected many important differentially expressed genes associated with P-deficiency tolerance by transcriptome analysis [26]. However, how DXWR copes with P-deficiency at the protein level is still unclear.

Label-free proteomics analysis is a method that can not only retain the authenticity of the sample to the greatest extent without relying on isotope labeling, but also can compare proteomes affected by different physiological conditions at the same time. Therefore, in this study, we used label-free proteomics analysis to detect the response of DXWR at the protein level under low P stress, and we combined it with the previous transcriptome data to further explore the low P tolerance genes in DXWR. These results would provide insights in explaining the molecular mechanism of low P resistance, as well as cloning and utilizing the P-deficiency genes from wild rice.

## 2. Materials and Methods

### 2.1. Plant Materials and Phosphorus Deficiency Treatment

In the present study, DXWR from Jiangxi academy of agricultural sciences was carried out as experimental material. The DXWR seeds were surface sterilized using mixed solutions of NaClO (10%) for 15min and soaked in petri dishes containing 20 mL deionized water at room temperature for 3 days. Then, the seeds were selected with the same growth trend and planted in the plastic pot containing quartz sand in a climate control chamber at day/night 14 h/10 h (30 °C/26 °C) [26]. A 1/2 Yoshida culture medium (pH 5.8) was added once for growth when germinated seeds had coleoptiles 10 mm approximately in length [27]. At the emergence of the third leaf (about 15 days), plants were transferred into either a culture medium with low P concentration (0.016 mM NaH_2_PO_4_) or normal P concentration (0.32 mM NaH_2_PO_4_), which corresponded to the -P treatments (RLP) and +P treatments (RCK), respectively. The culture medium was replaced every three days. There were 20 seedlings per treatment with three biological replications. Roots were harvested at 9 days after the experimental treatments started, and the samples were frozen immediately using liquid nitrogen and stored at −80 °C for further analyzing. Root samples of cultivated rice *Nipponbare* (NP) were obtained in the same way at the same time and used to determine the expression of *OsPHR2*, *OsPHR1*, *OsPHO2* (*Phosphate2*), and *OsPHO1* (*Phosphate Transporter 1*), as well as its natural reverse transcripts (*Cis-Natural Antisense Transcripts*, *NATs*).

### 2.2. Protein Extraction and Enzymatic Hydrolysis

Root proteins of six DXWR samples were extracted using trichloroacetic acid (TCA)-acetone precipitation method [28]. Briefly, the sample was ground into powder in liquid nitrogen and then suspended in extraction buffer (8 M urea, 1% DTT, 0.1 M Tris-HCl, pH 8.8, 1% complete protease inhibitors (Roche, Mannheim, Germany)). Repeated vortex of the sample and then removal of insoluble precipitation was performed by centrifugation at 14,000 ×g for 40 min. The supernatant was precipitated overnight with 20% (*v/v*) TCA, washed three times with cold acetone, and solubilized in extraction buffer. All operations were performed at low temperatures. The protein concentration was measured using the BCA Protein Assay Kit (Bio-Rad, Hercules, CA, USA). The final concentration of urea in protein solution was adjusted to 2 M with 40 mM NH_4_HCO_3_ solution. An amount of 4 μg trypsin (Promega, Madison, WI, USA) was added to each sample containing 200 μg protein and incubated overnight at 37 °C following the instructions of the manufacturer.

### 2.3. Liquid Chromatography and Tandem Mass Spectrometry Proteomics Analysis (LC-MS/MS) of the RLK and RCK Samples

LC-MS/MS analysis was performed on a Q-Exactive mass spectrometer (Thermo Fisher Scientific, Waltham, MA, USA) that was coupled to an Easy nLC Biosystem (Thermo Fisher Scientific, Waltham, MA, USA) with the help provided by Shanghai Applied Protein Technology (Shanghai, China). Balance chromatographic column with buffer A (0.1% formic acid, 3% acetonitrile and 97% H_2_O). Each sample was automatically injected into the prepacked column (2 cm × 100 µm 3 µm-C_18_), then flowed into an analytical column (10 cm × 75 µm 3 µm-C_18_) at a speed of 250 nL/min controlled by intelliflow technology. After that, the sample was separated with a linear gradient of buffer B from 6% (80% acetonitrile, 0.08% formic acid) to 95% over 116 min and then followed by an equilibration of the column at 6 % buffer B for 4 min. MS/MS spectra were searched using MaxQuant software (version 1.5.3.17) against the UniProt proteome database (Uniport_*Oryza sativa*_168264_20171201.fasta), and the label-free quantitation algorithm was performed for quantitative analysis. The maximum missed cleavages used for the database search were set to 2. The mass tolerance was set to 20 ppm on full scans. For label-free quantitative methods, retention time matching between runs was performed within a time window of 2 min. The peptide false discovery rate (FDR) and protein FDR did not exceed 0.01. This rigorous analysis tool named Andromeda was used for analysis-obtained excellent peptide score distribution to judge the quality of MS experimental data [29]. Quantifiable proteins were defined as those identified at least twice in the three biological replicates. Proteins with an adjusted *p* value < 0.05 were assigned as differentially expressed between the RLK and RCK samples [30].

### 2.4. Bioinformatics Analysis

With the help provided by Shanghai Applied Protein Technology (Shanghai, China), bioinformatics analysis was performed on the obtained proteome data. First, we searched the EBI database for conserved motifs that matched the target protein through InterProScan [31] and annotated the motif-related functional information to the target protein sequence to achieve gene ontology (GO) functional annotation [32,33]. Then, proteins were matched to the Kyoto encyclopedia of genes and genomes (KEGG) database to obtain the pathway they might participate in. Last but not least, proteome data set was used to construct protein-protein interaction (PPI) network by using STRING (https://cn.string-db.org/), and the parameter was set to moderate confidence (0.400).

### 2.5. Gene Expression Analysis by Quantitative Real-Time PCR (qRT-PCR)

For qRT-PCR analysis, total RNA in roots samples were isolated using TRIzol reagent (Invitrogen, Carlsbad, CA, USA) according to the manufacturer’s protocol. The first cDNA was synthesized with 2 μg of total RNA, using ReverTra Ace^®^ qPCR RT Master Mix with gDNA Remover (TOYOBO, Osaka, Japan). Briefly, RNA was pre-denatured at 65 °C, and gDNA was removed by adding DN Master Mix with gDNA Remover at 37 °C for 5 min. The first strand cDNA was synthesized by using RT Master Mix through the following three-step reaction: 37 °C for 15 min, 50 °C for 5 min, and 98 °C for 5 min. Then, synthesized cDNAs were used as templates for qRT-PCR with THUNDERBIRD™ SYBR^®^ qPCR Mix (TOYOBO, Osaka, Japan) and the LightCycler^®^ 96 instrument (Roche, Mannheim, Germany) according to the manufacturer’s manuals. The conditions of the qRT-PCR reaction are set as follows: 95 °C for 30 s with 1 cycle for pre-denaturation; two-step reaction with 40 cycles of 10 s at 95 °C and 30 s at 60 °C for amplification; three-step reaction for melt curve stage with 95 °C for 10 s, 65 °C for 60 s, and 97 °C for 1 s; 37 °C for 30 s for cooling. *OsActin1* (*LOC_Os03g50885*) was used as an internal control [34]. The 2^−∆∆Ct^ method was used for relative quantification. The statistical significance was evaluated by *t*-test analysis. The primers used are listed in Supplementary Table S1.

### 2.6. Conjoint Analysis of Proteomic and Transcriptomics Related to Low P Stress 

We selected the DXWR root transcriptome data obtained from samples treated at the same time with this study for comparison [26], and we screened the transcriptome data with the gene expression change fold ≥ 1.5 times and *p* value ≤ 0.05 after low P treatment for analysis. 

## 3. Results

### 3.1. Label-Free Quantitative Proteomic Analysis on DXWR

**Figure 1:**
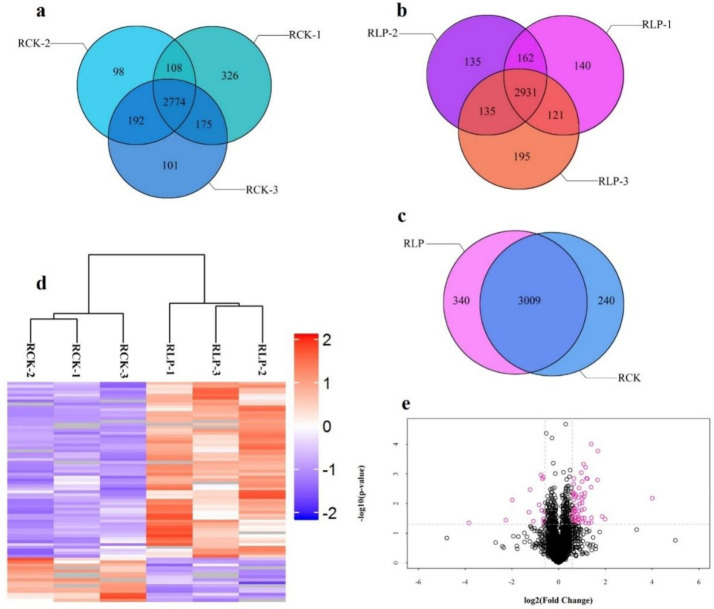
Identification and analysis of proteins that differentially accumulated between RCK and RLP. RLP, roots under low phosphorus stress treatment with three biological repetitions; RCK, roots under phosphorus sufficiency stress treatment with three biological repetitions, same to below. (**a**) Proteins identified in three RCK repeated materials. (**b**) Proteins identified in three RLP repeated materials. (**c**) Proteins identified in RCK and RLP. (**d**) Clustering analysis of proteins identified in RCK and RLP samples. (**e**) Volcano pot. The gene expression values were transformed to log2 scale. The protein expression fold change (X-axis) was plotted against the p value obtained from t test log10-value (Y-axis). Small circle represents protein. The red circle represents a protein with a change fold greater than 1.5.

By analyzing the peptide scores obtained by MS, the results showed that about 78.22% of the peptides scored above 60, which meant that the quality of LC-MS/MS experimental data was relatively high (Supplementary Figure S1). Through label-free proteomic data analysis, a total of 4329 protein groups (Supplementary Table S2) and 23,598 peptides (Supplementary Table S3) were identified from six DXWR root samples (RLP and RCK with three biological repetitions, respectively). Among these proteins, we designated 3589 proteins that were detected in at least two replicates as identified proteins (Figure 1a–c). In addition, the clustering analysis of proteins identified in different samples showed that the similarities between the three biological repetitions of the same treatment were high and between the different treatments were very low (Figure 1d). Based on this, it was indicated that changes in expression of these target proteins could represent a significant effect of biological treatment on samples. Using these data, we selected proteins with ≥ 1.5-fold changes as an additional standard [35], and the volcano pot was drawn by using protein expression fold changes and *p* value (Figure 1e). The results showed that 60 protein groups were up-regulated (Table 1) and 15 were downregulated (Table 2). Those results indicated that P-deficiency treatment could affect the accumulation of some gene expression products in DXWR.

### 3.2. Functional Classification by Gene Ontology (GO) 

**Figure 2:**
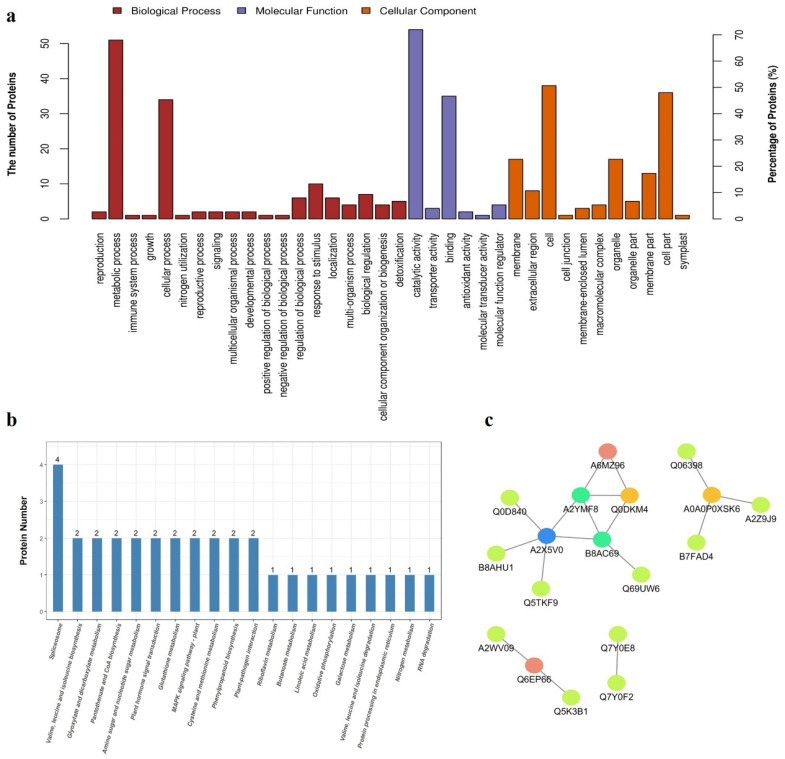
Analysis of identified proteins significantly different between the RCK and RLP samples. (**a**) Gene Ontology (GO) annotation of the proteins significantly different between the RCK and RLP samples. (**b**) The top 20 KEGG pathway assignments of the proteins significantly different between the RCK and RLP. The represented categories (*Q* ≤ 0.05) and the number of proteins predicted to belong to each category are shown. (**c**) The protein-protein interactions (PPI) between the identified proteins. The sphere represents the protein, and the straight line represents the interaction between the proteins at both ends of the straight line.

To gain insight into the functional roles of the proteins significantly different between the RCK and RLP samples, GO annotation and enrichment was conducted and the results were listed in Supplementary Table S4, schematically represented in three ontologies as molecular function, cellular component, and biological process, as in Figure 2a. The enrichment of biological process involved in metabolic process and cellular process was significantly observed. The most significantly enriched molecular function were catalytic activity and binding. Significant enrichment of cellular compartments was identified, including cell part, cell, membrane, membrane part, and organelle part. From the above description, we could give a conjecture that low P stress could affect cell proliferation and enzyme synthesis as well as the ability of cell or membrane to bind to certain stimulus signals in DXWR.

### 3.3. Kyoto Encyclopedia of Genes and Genomes (KEGG) Pathway Mapping

KEGG pathways analysis was performed on the 75 significantly different expression proteins (SDEPs, 60 up-regulated and 15 downregulated) identified in this study (Supplementary Table S5). These proteins were involved in 31 metabolic pathways. U6 snRNA-associated Sm-like protein 8 (LSm8, *LOC_Os05g51650*, up-regulated), U1 small nuclear ribonucleoprotein A (U1A, *LOC_Os05g06280*, up-regulated), splicing factor of arginine/serine-rich (SR, *LOC_Os01g06290*, downregulated), and pre-mRNA-splicing factor SPF27 (BCAS2, *LOC_Os01g16010*, downregulated) were enriched in the spliceosome pathway which was one of the top 20 KEGG pathways predicted to be affected by low P stress (Figure 2b). Furthermore, branched-chain-amino-acid aminotransferase (BCAT, *LOC_Os03g01600*, up-regulated) and acetolactate synthase small subunit (ALS, *LOC_Os11g14950*, up-regulated) were enriched in the pathway of branched-chain amino acids (BCAAs, including valine, leucine, and isoleucine) biosynthesis. Ribulose-1,5-bisphosphate carboxylase/oxygenase large subunit (rbcL, encoded by leucoplast, downregulated) and phosphoglycolate phosphatase (PGP, *LOC_Os09g08660*, up-regulated) were enriched in the pathway of glyoxylate and dicarboxylate metabolism. A chitinase (*LOC_Os01g49320*, up-regulated) and a glycosyl hydrolase (*LOC_Os01g47070*, up-regulated) were enriched in amino sugar and nucleotide sugar metabolism. In addition, two GST (*LOC_Os10g38740* and *LOC_Os10g38360*, up-regulated) proteins were enriched in the glutathione pathway. These results indicated that genes involved in these pathways might respond to low P stress in DXWR.

### 3.4. Protein-Protein Interaction (PPI) between the Low-P Responsive Proteins

Protein is an important component of biological organisms, which do not perform biological function independently, but through the interaction of proteins to regulate physiological and biochemical processes. Therefore, we performed a PPI analysis of the proteins identified by label-free quantitative analysis. As shown (Figure 2c), there was interaction only between a few proteins after screening. 

A2X5V0 (Uniprot ID, *LOC_Os02g33850*, up-regulated) with the function of elongation factor Tu family protein (EF-TU) has five mutual proteins, including Q0D840 (*LOC_Os07g08840*, up-regulated) with annotation of thioredoxin, A2YMF8 (*LOC_Os07g36490*, up-regulated) with annotation of RNA recognition, Q5TKF9 (*LOC_Os05g51650*, up-regulated) with annotation of U6 snRNA-associated Sm-like protein LSm8, B8AHU1 (*LOC_Os02g05880*, downregulated) with annotation of RNA polymerase, and B8AC69 (*LOC_Os01g16010*, downregulated) with annotation of BACS2 protein, which is the core component of the Prp19-related complex, along with being involved in important life activities such as splicing of precursor RNA. 

Additionally, B8AC69 and A2YMF8 both have four mutual proteins. Additionally, Q0DKM4 (*LOC_Os05g06280*, up-regulated) with annotation of U1 small nuclear ribonucleoprotein A and A0A0P0XSK6 (*LOC_Os10g07229*, up-regulated) with annotation of dehydrogenase have three mutual proteins, respectively. Q6EP66 (*LOC_Os09g08660*, up-regulated) with annotation of phosphoglycolate phosphatase and A6MZ96 (*LOC_Os01g06290*, downregulated) with annotation of splicing factor have two mutual proteins, respectively. These results suggested that low P stress induced reduction of transcription-related genes, and the increase of most RNA splicing related genes along with intensification of the gene expression associated with the elongation during translation.

### 3.5. Analysis of Differentially Expressed Proteins Responded to P-Deficiency in DXWR

**Figure 3:**
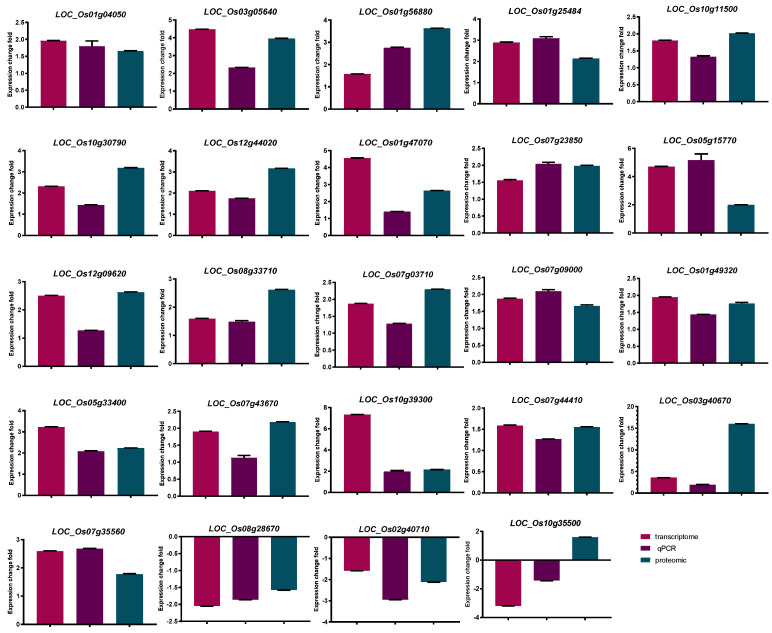
Quantitative real-time PCR analysis of 24 significant differential expression proteins with fold changes both in transcriptome and proteomics levels larger than 1.5 in DXWR. Bars mean SD. Expression change fold refers to the change of the treatment group compared with the control group.

In the present study, among 75 SDEPs, there were 24 proteins with fold changes larger than 1.5 both in proteomics and transcriptome level [26] that were associated with P-deficiency treatment in DXWR (Table 3). In addition, we also verified its expression at the transcriptome level by qRT-PCR, which showed consistency in transcriptome data and qRT-PCR results, shown in Figure 3. Among these proteins, 21 up-regulated and two downregulated at both transcriptome and proteome level, and only one had the opposite abundance trend (up-regulated in proteome level but downregulated in transcriptome level). There were some genes whose expression abundance increased in both protein and transcription levels, including *OsPT2* (*LOC_Os03g05640*), *OsPT8* (*LOC_Os10g30790*), *OsPAP10c* (*LOC_Os12g44020*), *OsPAP10a* (*LOC_Os01g56880*), *OsPHF1* (*LOC_Os07g09000*), as well as a gene encoding glycerophosphoryl diester phosphodiesterase family protein (GDPD, *LOC_Os03g40670*), three genes encoding glycosyl hydrolase (*LOC_Os07g23850*, *LOC_Os05g15770* and *LOC_Os01g47070*), and one gene encoding glucan endo-1,3-beta-glucosidase precursor (*LOC_Os07g35560*). Furthermore, a gene (*LOC_Os10g35500*) encoding epoxide hydrolase increased by low P stress at proteomic level, but the corresponding transcription levels decreased. The above results indicated that after low P stress treatment, there would be differences in the trend and degree of change in gene expression at the transcription and translation levels. 

### 3.6. Conjoint Analysis of Proteomic and QTLs Related to P-Deficiency Tolerance 

As shown in Table 4, there are two P-deficiency tolerance related QTLs have been identified in DXWR [23], and 12 QTLs existing in different positions on the chromosome related to P-deficiency stress have been found in *Oryza sativa* based on the Gramene QTL database. Among genes corresponding to 75 SDEPs identified by the proteome in this study, we located nine genes among these QTL intervals, as shown in Table 5. Among them, two genes (*LOC_Os12g44020*, *OsPAP10c* and *LOC_Os04g41970*, *OsGLU3*) have been characterized in previous studies [36,37], and two of the other seven uncharacterized genes (*LOC_Os12g09620* and *LOC_Os03g40670*) have been detected at both transcriptome and proteome levels. Furthermore, the functional expression characteristics of the remaining five genes (*LOC_Os01g57450*, *LOC_Os03g29240*, *LOC_Os03g13540*, *LOC_Os03g29190* and *LOC_Os06g07600*) have not been reported yet.

### 3.7. The Expression Pattern of Genes Related to P-Deficiency Tolerance in DXWR 

**Figure 4:**
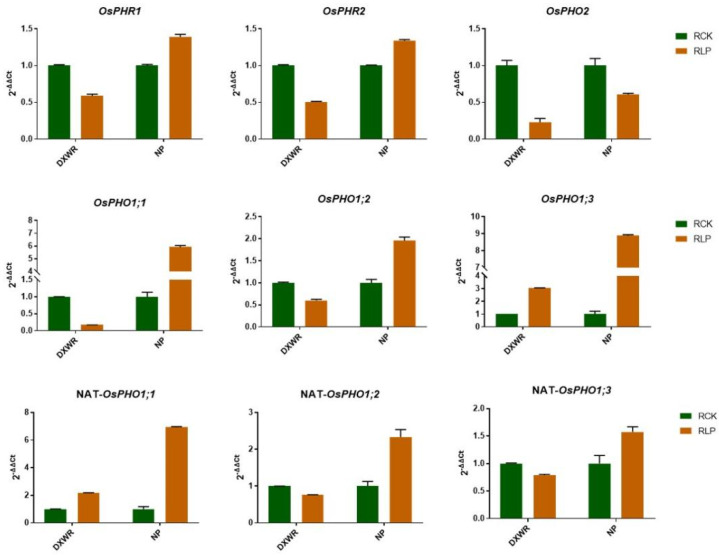
Expression pattern in DXWR and NP of key genes that characterized in cultivated rice participating in the P-regulation network detected by qRT-PCR. Error bar means standard deviation.

Based on previous studies on P-response mechanism in cultivated rice, the homologous genes of *PHR1* (*OsPHR2* and *OsPHR1*), *OsPHO2*, *OsPHO1,* as well as its NATs, play an important role in the low P-response process [7,38]. Therefore, we examined the expression levels of *OsPHR2* and *OsPHO2* as well as three members of *OsPHO1* (*OsPHO1;1*, *OsPHO1;2* and *OsPHO1;3*) and three NATs correspondently, as shown in Figure 4. We found that *OsPHR2* was downregulated after low P treatment in DXWR roots, which was contrary to the results of previous studies on cultivated rice [7,38]. We suspect that *OsPHR1* may play a major role during the P starvation signaling pathway in DXWR. For validation, we examined the expression of *OsPHR1* in DXWR, but the result was consistent with *OsPHR2* and also down-regulated. Such results may indicate that the transcription levels of *OsPHR1* and *OsPHR2* are inhibited by low P signals in DXWR. In addition, *OsPHO2* was down-regulated in both DXWR and NP. Among *OsPHO1;1*, *OsPHO1;2*, and *OsPHO1;3*, only *OsPHO1;3* expression was up-regulated and the other two were downregulated in DXWR, but three genes all up-regulated in NP. In the corresponding NAT, only *OsPHO1;2* NAT expression change trend is consistent with *OsPHO1;2*, and the other two NATs change trend is opposite to *OsPHO1;1* and *OsPHO1;3* in DXWR. However, all three NATs up-regulated in NP. These results indicate that there may be a difference in the mechanism of resistance to low P in DXWR compared to cultivated rice.

## 4. Discussion 

### 4.1. Low P Stress Leads to Differential Expression of P Absorption Efficiency Related Genes in DXWR

A previous study [39] has shown that OsPHF1 regulates the plasma membrane localization of low-affinity Pi transporter *OsPT2* and the high-affinity Pi transporter *OsPT8*, of which ortholog in *Arabidopsis* reported to be only an important factor for the localization of high-affinity Pi transporters to the plasma membrane [40]. Subcellular location experiments show that mutation of *OsPHF1* lead to the retention of *OsPT2* and *OsPT8* in the endoplasmic reticulum and reduce the accumulation of Pi in shoots due to overexpression of *OsPHR2* [39,41]. To the contrary, overexpression of *OsPHF1* results in excessive Pi accumulation in leaf and root. Furthermore, *OsPT2* is the only low affinity transporter in the *PHT1* family induced by Pi deprivation under the transcriptional control of OsPHR2, whereas *OsPT8* is constitutively expressed high-affinity Pi transporters in rice whose expression is not affected by external Pi levels. In our study, the expression levels of *OsPT2* (*LOC_Os03g05640*), *OsPT8* (*LOC_Os10g30790*) and *OsPHF1* (*LOC_Os07g09000*) were up-regulated. The up-regulated expression of *OsPHF1* may increase the plasma membrane localization of OsPT2 and OsPT8. 

On the other hand, purple acid phosphatase (PAP) is a family of metals phosphoesterases involved in a variety of physiological functions, especially in low Pi adaptations in plants [42]. PAPs have non-specific acidic phosphatase activity, which can catalyze the hydrolysis of various organic P into Pi under acidic pH conditions, thus providing more Pi for plants [36,43]. PAP plays a critical role in the plant’s ability to utilize organic P in growth medium. There were two PAP genes, *OsPAP10c* (*LOC_Os12g44020*) and *OsPAP10a* (*LOC_Os01g56880*) induced by low P stress in DXWR. It is likely that they play a crucial role in the ability of plants to use organic P.

In addition to the above-mentioned genes that may increase the efficiency of P absorption of DXWR, there are other functional genes that are differentially expressed. Studies have shown that PGP inactivation attenuated triosephosphate isomerase activity, thereby increasing triglyceride levels at the expense of the cellular phosphatidylcholine content, and inhibiting cell proliferation [44,45]. These effects were prevented under hypoxic conditions or by blocking phosphoglycolate release from damaged DNA. Moreover, as shown, EF-TU plays an important role in the reproduction, development, and response to environmental stress of higher plants [46]. Here, the increased synthesis of PGP (*LOC_Os09g08660*) and EF-TU (*LOC_Os02g33850*) may be the response of DXWR to low P stress. Furthermore, chitinase leads to the separation of parallel chitin microfibrils connected by β-1,6-branched chain β-1,3- glucans in the cell wall, thus increasing the interval between the insertion of newly synthesized chitin and β-1,3- glucans under swelling *in vivo* [47]. In this study, it was worth noting that the expression of chitinase (*LOC_Os01g49320*) and glycosyl hydrolase (*LOC_Os01g47070*) increased when the root of DXWR was under low P stress. The differential expression of these genes may imply a strategy for DXWR to respond to low P stress by improving P absorption efficiency.

### 4.2. Differential Expression of Variable Splicing-Related Genes May Contribute to the Low P Resistance for DXWR

Studies have shown that the absence of SR or SPF protein can lead to changes in splice sites [48,49]. LSm8 is essential for the assembly of the LSM nuclear complex (LSm2-8) and this complex acts in pre-mRNA splicing through U6 snRNA stabilization, thus allowing the formation of the U6 snRNP [50]. The *Arabidopsis* LSM2-8 complex differentially regulates plant tolerance to abiotic stresses by controlling the constitutive and alternative splicing of specific introns from selected abiotic stress-related pre-mRNAs [51]. In this study, the up-regulation of LSm8 (*LOC_Os05g51650*) and U1A (*LOC_Os05g06280*) may alternate splicing of pre-mRNA, while the down-regulation of SPF (*LOC_Os01g16010*) and SR (*LOC_Os01g06290*) may change the pre-mRNA splicing site, thus contributing to low P resistance of DXWR.

### 4.3. Increase the Antioxidant Capacity of DXWR by Regulating the Expression of Related Genes

Studies have shown that plants under abiotic stress are coerced to increase the activity of reactive oxygen species (ROS) and antioxidant enzymes [52,53]. As shown, a key enzyme in the BCAAs biosynthesis pathway, ALS, was downregulated under stress [52]. In addition, by increasing the concentration of BCAAs to 100 mg/L in the culture medium, ROS was significantly reduced, thereby reducing the level of antioxidant enzymes in herbicide-stressed plants [53]. In present research, DXWR may enhance the biosynthesis of BCAAs through up-regulating BCAT (*LOC_Os03g01600*) and ALS (*LOC_Os11g14950*) to reduce ROS and consequently antioxidant enzyme levels. What′s more, Glutathione is a co-substrate for glutathione-S-transferase (GST), which in rice participates in various functions such as phytohormone homeostasis, hydroxy peroxide detoxification, apoptosis regulation [54], and also has a key role in response to biotic and abiotic stresses [55]. In this study, two GST proteins (*LOC_Os10g38740* and *LOC_Os10g38360*) enriched in glutathione metabolism pathway were up-regulated after P-deficiency treatment of DXWR which might increase the tolerance to cope with low P stress. Furthermore, epoxide hydrolase has been reported as an enzyme that reduces the content of epoxides in organisms by means of chemically catalyzed transformation, as well as metabolizes endogenous aliphatic and aromatic epoxides [56]. It is a key enzyme involved in metabolism, detoxification, and signaling regulation in the organism [57]. In this study, an epoxide hydrolase protein (*LOC_Os10g35500*) increased in protein level might increase the tolerance to low P stress, but the corresponding transcription level decreased, possibly by increasing the stability of the corresponding mRNA and reducing the number of transcriptions to reduce energy consumption.

### 4.4. DXWR May Exist a Low P Tolerance Mechanism Different from Cultivated Rice NP 

PHR1 is a central regulator of P-deficiency stress response [6]. There are two homologous genes of *PHR1* in rice, namely *OsPHR1* and *OsPHR2*. Some studies have shown that *OsPHR2* plays a major role in the P starvation signaling pathway that is induced by low phosphorus stress. [7,38]. However, in this study, both *OsPHR2* and *OsPHR1* were downregulated after receiving low P stress in DXWR, which was opposite to that in NP. After Pi enters the column cells of the root, PHO1 located in the xylem of the root vascular bundle is responsible for loading Pi into the xylem and then transporting it from the xylem to the shoot [58,59]. In *Arabidopsis*, mainly *AtPHO1* and *AtPHO1;H1* are involved in the P transportation from root to shoot [60]. There are three homologous genes of *AtPHO1* and *AtPHO1;H1* in rice, which are *OsPHO1;1*, *OsPHO1;2,* and *OsPHO1; 3*, and all three genes have NAT at the 5’ end [38]. Studies among cultivated rice showed that *OsPHO1;2* played a main role in the transport and distribution of P, while *OsPHO1;2* NAT was induced by P starvation and could activate the expression of *OsPHO1;2* [38], which was consistent with our results in NP. However, in DXWR, *OsPHO1;2* and its NAT both downregulated by low P stress, whereas *OsPHO1;1* downregulated and its NAT up-regulated, as well as *OsPHO1;3* up-regulated and its NAT downregulated. In addition, OsPHO2 containing a E2 ubiquitin-binding domain could degrade OsPHO1 [61], of which the downregulated expression results consistent with previous studies were obtained in both DXWR and NP. Through the above analysis, we found that *OsPHR1 OsPHR2*, three *OsPHO1,* and the corresponding *NATs* showed different response trends in DXWR and NP, except for *OsPHO2*. These differences indicate that DXWR may have a unique resistance to low P regulation, and these candidate genes screened by transcriptome and proteome in the present study may also have unique functions in DXWR different from cultivated rice.

## 5. Conclusions

In this study, label-free proteomics analysis as well as joint analysis with transcriptome dataset were conducted to root samples to identify potential unique low P-response genes in DXWR during seedlings. 75 SDEPs were detected, 24 of which were also detected in previous transcriptome dataset verified by qRT-PCR. Furthermore, it was found that DXWR could increase PAPs’ expression, membrane location of PTs, rhizosphere area, alternative splicing and decrease ROS activity to deal with low P stress. Moreover, among the genes corresponding to 75 SDEPs, seven uncharacterized genes were located in previous P related QTL intervals, of which two genes (*LOC_Os12g09620* and *LOC_Os03g40670*) have been detected at both transcriptome and proteome levels. In addition, the expression patterns of *OsPHR1*, *OsPHR2*, *OsPHO1*, and *NAT-OsPHO1* in DXWR were different in cultivated rice NP, suggesting that the response mechanism of some low P tolerance in DXWR might be different from that in cultivated rice. These findings would provide insights in cloning the P-deficiency genes from wild rice, as well as elucidating the molecular mechanism of low P resistance in DXWR.

## Figures and Tables

**Table 1:** Up-regulated proteins identified from label-free quantitative analysis.

RAP (Os ID)	MSU (LOC_Os ID)	Description	Gene Name	Ratio (RLP/RCK)	*p* Value
*Os03g0603600*	*LOC_Os03g40670*	Glycerophosphoryl diester phosphodiesterase family protein	*OSJNBa0004G03.4*	16.03000419	0.006552
*Os03g0150800*	*LOC_Os03g05640*	Inorganic phosphate transporter 1-2	*PTH1-2*	3.960889785	0.033385
*Os01g0776600*	*LOC_Os01g56880*	Purple acid phosphatase	*Os01g0776600*	3.622714483	0.02748
*Os10g0444700*	*LOC_Os10g30790*	Probable inorganic phosphate transporter 1–8	*PHT1-8*	3.188357926	0.000168
*Os12g0637100*	*LOC_Os12g44020*	Purple acid phosphatase	*LOC_Os12g44020*	3.158117784	0.001597
*Os01g0660200*	*LOC_Os01g47070*	Glycosyl hydrolase	*OsJ_02893*	2.640670999	0.030145
*Os03g0719300*	*LOC_Os03g51000*	3,4-dihydroxy-2-butanone kinase	*B1377B10.5*	2.626305612	0.0442
*Os12g0198000*	*LOC_Os12g09620*	Expressed protein	*Os12g0198000*	2.624379146	9.82 × 10^−5^
*Os08g0434100*	*LOC_Os08g33710*	Ribonuclease		2.61471967	0.002771
*Os06g0291100*	*LOC_Os06g18790*	Anthocyanidin 3-O-glucosyltransferase	*B1026E06.27*	2.435586728	0.046309
*Os02g0704900*	*LOC_Os02g47600*	Soluble inorganic pyrophosphatase	*IPP*	2.329695866	0.001458
*Os07g0129200*	*LOC_Os07g03710*	SCP-like extracellular protein	*OsJ_22957*	2.29462251	0.001414
*Os05g0402900*	*LOC_Os05g33400*	Basic 7S globulin precursor	*OsJ_18488*	2.229986706	0.045047
*Os03g0405900*	*LOC_Os03g29240*	Probable nucleoredoxin 1-2	*Os03g0405900*	2.22151414	0.0006
*Os07g0630400*	*LOC_Os07g43670*	Ribonuclease T2 family domain containing protein	*P0011H09.133*	2.18513976	0.004417
*Os10g0538200*	*LOC_Os10g39300*	Aspartic proteinase nepenthesin, putative, expressed	*OsI_34482*	2.147759239	0.030829
*Os01g0357100*	*LOC_Os01g25484*	Ferredoxin-nitrite reductase	*OsI_01871*	2.139517104	0.015234
*Os11g0256050*	*LOC_Os11g14950*	Acetolactate synthase small subunit	*LOC_Os11g14950*	2.118353002	0.005821
*Os02g0543300*	*LOC_Os02g33850*	Elongation factor Tu family protein, Protein synthesis factor, GTP-binding domain containing protein	*OsI_07585*	2.087060146	0.000463
*Os07g0549800*	*LOC_Os07g36490*	RNA recognition motif containing protein	*OsI_26412*	2.035407774	0.042675
*Os03g0738600*	*LOC_Os03g52860*	Linoleate 9S-lipoxygenase 2	*LOX1.1*	2.017839574	0.006819
*Os10g0191300*	*LOC_Os10g11500*	SCP-like extracellular protein	*LOC_Os10g11500*	2.010712464	0.0018
*Os05g0247100*	*LOC_Os05g15770*	Glycoside hydrolase family 18	*dip3*	2.004889132	0.03089
*Os10g0476000*	*LOC_Os10g33630*	Adaptin ear-binding coat-associated protein 2	*Os10g0476000*	1.997085229	0.009577
*Os04g0480900*	*LOC_Os04g40490*	Glycosyl hydrolase family 5 protein	*OsI_16340*	1.983137882	0.00484
*NONE*	*LOC_Os07g23850*	Glycosyl hydrolase	*OsI_25770*	1.977235788	0.016379
*Os03g0238600*	*LOC_Os03g13540*	Purple acid phosphatase	*LOC_Os03g13540*	1.955506958	0.0009
*Os06g0172800*	*LOC_Os06g07600*	Uncharacterized glycosyltransferase	*OsJ_20295*	1.871718049	0.010126
*Os01g0949900*	*LOC_Os01g72150*	Glutathione S-transferase	*Os01g0949900*	1.826722973	0.002976
*Os03g0405500*	*LOC_Os03g29190*	Probable nucleoredoxin 1-1	*Os03g0405500*	1.822377514	0.017644
*Os07g0162700*	*LOC_Os07g06860*	Gibberellin receptor GID1L2	*P0428D12.107*	1.818251432	0.034418
*Os01g0747500*	*LOC_Os01g54370*	Dihydropyrimidinase	*OsI_03720*	1.785266614	0.046646
*Os06g0320000*	*LOC_Os06g21550*	Thioredoxin-like protein Clot	*Os06g0320000*	1.776329132	0.032953
*Os07g0658600*	*LOC_Os07g46480*	Eukaryotic aspartyl protease domain containing protein	*OsJ_25435*	1.774156573	0.006259
*Os07g0539900*	*LOC_Os07g35560*	Glucan endo-1,3-beta-glucosidase precursor	*OsJ_24595*	1.773462618	0.027652
*Os01g0687400*	*LOC_Os01g49320*	Chitinase		1.760766916	0.04272
*Os02g0771700*	*LOC_Os02g53200*	Glucan endo-1,3-beta-glucosidase precursor	*Os02g0771700*	1.735448414	0.022941
*Os07g0186000*	*LOC_Os07g08840*	Thioredoxin H1	*TRXH*	1.701278791	0.011916
*Os04g0456700*	*LOC_Os04g38390*	Wound/stress protein, putative, expressed	*OSJNBa0036B21.4*	1.667066756	0.004973
*Os07g0187700*	*LOC_Os07g09000*	WD40 protein, regulation of the plasma membrane localization of phosphate transporters, phosphate uptake and translocation	*Os07g0187700*	1.663966106	0.037637
*Os01g0132000*	*LOC_Os01g04050*	BBTI12-Bowman-Birk type bran trypsin inhibitor precursor	*Os01g0132000*	1.654255218	0.017942
*Os07g0683600*	*LOC_Os07g48460*	Stress responsive protein, putative, expressed	*OsJ_25614*	1.644255664	0.005868
*Os03g0106400*	*LOC_Os03g01600*	Branched-chain-amino-acid aminotransferase	*LOC_Os03g01600*	1.610695927	0.030361
*Os10g0498100*	*LOC_Os10g35500*	Epoxide hydrolase	*OsJ_32041*	1.592639094	0.014471
*Os06g0717900*	*LOC_Os06g50390*	Aspartic proteinase nepenthesin II-like	*P0541C02.19-1*	1.585853919	0.014796
*Os07g0634600*	*LOC_Os07g44070*	Pectin acetylesterase	*P0455H11.118-1*	1.583424242	0.017612
*Os09g0261300*	*LOC_Os09g08660*	Phosphoglycolate phosphatase	*B1077E10.18-1*	1.57844691	0.004606
*Os04g0628200*	*LOC_Os04g53640*	Peroxidase	*prx56*	1.572207357	0.001422
*Os10g0527800*	*LOC_Os10g38360*	Glutathione S-transferase	*OsI_34399*	1.558245126	0.045511
*Os05g0154800*	*LOC_Os05g06280*	U1 small nuclear ribonucleoprotein A	*Os05g0154800*	1.55665498	0.007471
*Os03g0661600*	*LOC_Os03g45960*	Similar to Alpha-amylase/trypsin inhibitor (Antifungal protein).	*OSJNBb0065L20.2*	1.553387114	0.019769
*Os03g0214000*	*LOC_Os03g11530*	Purple acid phosphatase	*LOC_Os03g11530*	1.550828031	0.035302
*Os07g0638100*	*LOC_Os07g44410*	WD40-like Beta Propeller Repeat family protein	*OJ1340_C08.105*	1.550189963	0.002186
*Os10g0530900*	*LOC_Os10g38740*	Probable glutathione S-transferase GSTU6	*GSTU6*	1.530424487	0.014539
*Os01g0783500*	*LOC_Os01g57450*	Universal stress protein domain containing protein	*Os01g0783500*	1.526457541	0.005096
*Os10g0159800*	*LOC_Os10g07229*	dehydrogenase	*Os10g0159800*	1.524049105	0.049332
*Os02g0139100*	*LOC_Os02g04650*	Activator of Hsp90 ATPase	*Os02g0139100*	1.511733905	0.004122
*Os06g0266400*	*LOC_Os06g15600*	Similar to chemocyanin Phytocyanin	*OsI_22465*	1.508518489	0.04803
*Os05g0182100*	*LOC_Os05g08930*	chloroplast lumen common family protein	*OsI_18722*	1.500572906	0.021029
*Os05g0594900*	*LOC_Os05g51650*	U6 snRNA-associated Sm-like protein LSm8	*Os05g0594900*	1.50032122	0.029511

**Table 2:** Downregulated proteins identified from label-free quantitative analysis.

RAP (Os ID)	MSU (LOC_Os ID)	Description	Gene Name	Ratio (RLP/RCK)	*p* Value
*Os02g0822800*	*LOC_Os02g57690*	Acyl-CoA binding protein-like	*Os02g0822800*	0.657506509	0.019206616
*Os03g0219200*	*LOC_Os03g11960*	copper/zinc superoxide dismutase	*Os03g0219200*	0.637308003	0.049986265
*Os08g0374000*	*LOC_Os08g28670*	Bet v I allergen family protein	*Os08g0374000*	0.636556967	0.001251447
*Os06g0104300*	*LOC_Os06g01490*	monocopper oxidase	*Os06g0104300*	0.636141093	0.039078829
*Os01g0155600*	*LOC_Os01g06290*	Splicing factor, arginine/serine-rich		0.635942121	0.032973117
*Os08g0441500*	*LOC_Os08g34280*	Cinnamoyl-CoA reductase, lignin formation	*P0528B09.35-1*	0.617419753	0.001449421
*Os05g0278500*	*LOC_Os05g19910*	Acyl transferase 5	*AT5*	0.606305592	0.016918153
*Os05g0135700*	*LOC_Os05g04510*	S-adenosylmethionine synthase, catalyzes the formation of S-adenosylmethionine from methionine and ATP.	*sams*	0.59241418	0.001074265
*Os05g0375400*	*LOC_Os05g31140*	Glucanase	*GLU*	0.56122895	0.010900711
*Os02g0620500*	*LOC_Os02g40710*	Ammonium transporter 1 member 3	*AMT1-3*	0.473226337	0.03929424
*Os01g0717700*	*LOC_Os01g52010*	alliin lyase precursor	*Os01g0717700*	0.428345703	0.003389562
*Os04g0497200*	*LOC_Os04g41970*	Endoglucanase 12	*GLU3*	0.414420949	0.019558331
*Os01g0264900*	*LOC_Os01g16010*	BCAS2 protein, putative, expressed	*OsI_01292*	0.253067847	0.00757617
	cpDNA	ribulose-1,5-bisphosphate carboxylase/oxygenase large subunit, RuBisCO	*rbcL*	0.208373843	0.03601915
*Os02g0152700*	*LOC_Os02g05880*	DNA-directed RNA polymerase subunit	*OsI_05888*	0.069922407	0.045181841

**Table 3:** Significant differential expression proteins (*p* ≤ 0.05) with fold changes both in transcriptome and proteomics level larger than 1.5.

RAP (Os ID)	MSU (LOC_Os ID)	RLP/RCK in Transcriptome	RLP/RCK in Proteomic	*p* Value	Protein IDs	Annotation
*Os03g0603600*	*LOC_Os03g40670*	3.572344	16.03	0.006552	Q6AUZ6	Glycerophosphoryl diester phosphodiesterase family protein, expressed
*Os03g0150800*	*LOC_Os03g05640*	4.470767	3.96089	0.033385	Q8GSD9	Low-affinity transporter for inorganic phosphate (Pi)
*Os01g0776600*	*LOC_Os01g56880*	1.57171	3.622714	0.02748	A0A0P0V8Z3	Purple acid phosphatase
*Os10g0444700*	*LOC_Os10g30790*	2.315579	3.188358	0.000168	Q8H6G8	Probable inorganic phosphate transporter 1-8
*Os12g0637100*	*LOC_Os12g44020*	2.109139	3.158118	0.001597	Q2QLL9	Purple acid phosphatase
*Os01g0660200*	*LOC_Os01g47070*	4.556587	2.640671	0.030145	A2ZW76	Glycosyl hydrolase
*Os12g0198000*	*LOC_Os12g09620*	2.500715	2.624379	9.82E-05	Q2QWE5	Expressed protein
*Os08g0434100*	*LOC_Os08g33710*	1.591087	2.61472	0.002771	Q9FRU0	Ribonuclease
*Os07g0129200*	*LOC_Os07g03710*	1.872105	2.294623	0.001414	B9FVB5	SCP-like extracellular protein, expressed
*Os05g0402900*	*LOC_Os05g33400*	3.22764	2.229987	0.045047	B9FPI6	Basic 7S globulin precursor, putative, expressed
*Os07g0630400*	*LOC_Os07g43670*	1.90585	2.18514	0.004417	Q8H4E4	Ribonuclease T2 family domain containing protein, expressed
*Os10g0538200*	*LOC_Os10g39300*	7.334062	2.147759	0.030829	A2Z9R9	Aspartic proteinase nepenthesin, putative, expressed
*Os01g0357100*	*LOC_Os01g25484*	2.89252	2.139517	0.015234	B8A7W8	Ferredoxin--nitrite reductase, putative, expressed
*Os10g0191300*	*LOC_Os10g11500*	1.800917	2.010712	0.0018	Q8LMW8	SCP-like extracellular protein, expressed
*Os05g0247100*	*LOC_Os05g15770*	4.70642	2.004889	0.03089	Q5WMX0	Similar to glycosyl hydrolases Family 18
*NONE*	*LOC_Os07g23850*	1.549946	1.977236	0.016379	A2YKM4	Glycosyl hydrolase
*Os07g0539900*	*LOC_Os07g35560*	2.59231	1.773463	0.027652	B9FXQ1	Glucan endo-1,3-beta-glucosidase precursor, putative, expressed
*Os01g0687400*	*LOC_Os01g49320*	1.944762	1.760767	0.04272	Q7XXQ0	Chitinase
*Os07g0187700*	*LOC_Os07g09000*	1.874014	1.663966	0.037637	Q6Z4F3	WD40 protein, regulation of the plasma membrane localization of phosphate transporters, Phosphate uptake and translocation
*Os01g0132000*	*LOC_Os01g04050*	1.955063	1.654255	0.017942	Q9LGB2	BBTI12 - Bowman-Birk type bran trypsin inhibitor precursor, expressed
*Os10g0498100*	*LOC_Os10g35500*	0.314414	1.592639	0.014471	A3C655	Epoxide hydrolase
*Os07g0638100*	*LOC_Os07g44410*	1.589194	1.55019	0.002186	Q8GVH2	WD40-like Beta propeller repeat family protein
*Os08g0374000*	*LOC_Os08g28670*	0.489589	0.636557	0.001251	Q6ZD29	Bet v I allergen family protein OsBet v I
*Os02g0620500*	*LOC_Os02g40710*	0.635764	0.473226	0.039294	Q6K9G3	Ammonium transporter 1 member 3

**Table 4:** Previously identified P-deficiency responses related to QTL intervals.

QTL ID	Species Name	Chromosome	Position
AQBD004	*Oryza sativa*	1	41,967,890–41,969,197 bp
AQCI001	*Oryza sativa*	2	8,984,645–18,496,476 bp
AQCI008	*Oryza sativa*	3	6,753,341–10,322,897 bp
AQCI006	*Oryza sativa*	4	88,362–4,439,573 bp
AQCI011	*Oryza sativa*	4	24,690,120–27,908,404 bp
AQCI002	*Oryza sativa*	6	3,536,009–4,952,592 bp
AQCI009	*Oryza sativa*	6	1,644,474–4,952,592 bp
AQCI003	*Oryza sativa*	10	7,639,733–14,271,753 bp
AQBD007	*Oryza sativa*	12	1,548,040–1,548,464 bp
AQCI012	*Oryza sativa*	12	3,885,926–27,489,485 bp
AQCI013	*Oryza sativa*	12	1,548,040–18,867,702 bp
AQAZ001	*Oryza sativa*	12	13,101,084–15,120,848 bp
qMLR-1	DXWR	1	33,053,493–36,734,272 bp
qTDW-2	DXWR	3	12,407,382–23,822,102 bp

bp = base pair.

**Table 5:** Located genes encoded significantly different expression proteins identified from label-free quantitative analysis among previously identified P-deficiency responses related QTL intervals.

RAP (Os ID)	MSU (LOC_Os ID)	Mapped QTL Accession ID	Description	Ratio (RLP/RCK)	*p* Value
*Os01g0783500*	*LOC_Os01g57450*	qMLR-1	Universal stress protein domain containing protein	1.52646	0.005096
*Os03g0603600*	*LOC_Os03g40670*	qTDW-2	Glycerophosphoryl diester phosphodiesterase family protein	16.03	0.006552
*Os03g0405900*	*LOC_Os03g29240*	qTDW-2	Probable nucleoredoxin 1-2	2.22151	0.0006
*Os03g0238600*	*LOC_Os03g13540*	AQCI008	Purple acid phosphatase	1.95551	0.0009
*Os03g0405500*	*LOC_Os03g29190*	qTDW-2	Probable nucleoredoxin 1-1	1.82238	0.017644
*Os06g0172800*	*LOC_Os06g07600*	AQCI002, AQCI009	Uncharacterized glycosyltransferase	1.87172	0.010126
*Os12g0637100*	*LOC_Os12g44020*	AQCI012	Purple acid phosphatase	3.15812	0.001597
*Os12g0198000*	*LOC_Os12g09620*	AQCI012, AQCI013	Expressed protein	2.62438	9.82 × 10^−5^
*Os04g0497200*	*LOC_Os04g41970*	AQCI011	Endoglucanase 12	0.41442	0.019558331

## Data Availability

Data is contained within the article and Supplementary Material.

## Supplementary Materials

The following are available online at https://www.mdpi.com/article/10.3390/genes13010108/s1, Supplementary Table S1: Primer used for qRT-PCR analysis. Supplementary Figure S1: Molecular weight distribution. Supplementary Table S2: List of proteins identified from RLP and RCK samples with three biological repetitions, respectively. Supplementary Table S3: List of peptides identified from RLP and RCK samples with three biological repetitions, respectively. Supplementary Table S4: List of GO function enrichment analysis of proteins identified from RLP and RCK. Supplementary Table S5: List of KEGG pathway mapping of proteins identified from RLP and RCK.

## Abbreviations

P	phosphorus
DXWR	Dongxiang wild rice
SDEPs	significantly different expression proteins
PTs	P transporters
P1BS	PHR1 Binding Sequence
GO	gene ontology
KEGG	kyoto encyclopedia of genes and genomes
LSm8	U6 snRNA-associated Sm-like protein 8
U1A	U1 small nuclear ribonucleoprotein A
BCAT	branched-chain-amino-acid aminotransferase
ALS	acetolactate synthase small subunit
rbcL	ribulose-1,5-bisphosphate carboxylase/oxygenase large subunit
PGP	phosphoglycolate phosphatase
PPI	protein-protein interactions
EF-TU	elongation factor Tu family protein
GDPD	glycerophosphoryl diester phosphodiesterase family protein
PAP	purple acid phosphatases;
GST	glutathione-S-transferase
PHR1	phosphate starvation response regulator 1
qRT-PCR	quantitative real-time PCR
