# Supplementary material for: Identification of Phosphorus Stress Related Proteins in the Seedlings of Dongxiang Wild Rice (Oryza Rufipogon Griff.) Using Label-Free Quantitative Proteomic Analysis
Source: Genes (Basel). 2022 Jan 4;13(1):108. doi: 10.3390/genes13010108 (PMC8774503; doi:10.3390/genes13010108)
Supplement: Supplementary file 1 [file genes-13-00108-s001.zip › Supplementary Figure S1.pdf]

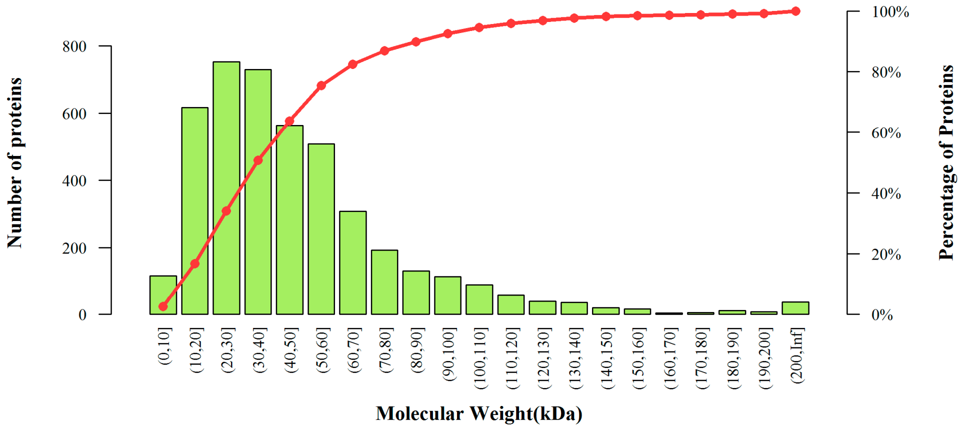

Supplementary Figure S1. Molecular weight distribution

The abscissa is the relative molecular weight of the identified protein; the primary ordinate-Number of proteins corresponds to the histogram in the graph, indicating the number of identified proteins with the corresponding relative molecular mass; the secondary ordinate corresponds to the cumulative curve in the graph, indicating the cumulative percentage of proteins that have no higher than the corresponding relative molecular mass.
