# Supplementary material for: Identification of Phosphorus Stress Related Proteins in the Seedlings of Dongxiang Wild Rice (Oryza Rufipogon Griff.) Using Label-Free Quantitative Proteomic Analysis
Source: Genes (Basel). 2022 Jan 4;13(1):108. doi: 10.3390/genes13010108 (PMC8774503; doi:10.3390/genes13010108)
Supplement: Supplementary file 1 [file genes-13-00108-s001.zip › Supplementary Table S1.pdf]

Supplementary Table S1. Primer used for qRT-PCR analysis

Primer name	Sequence
LOC_Os01g56880-F	ATCAAGAACAGGACGCACGC
LOC_Os01g56880-R	GAACCACATGGAGTCAGCCG
LOC_Os03g51000-F	TCGCGGTTATCTCAGGTGGT
LOC_Os03g51000-R	CGGCAGCTGTCAACATTCCT
LOC_Os08g33710-F	GTCACTGACACGTGGGTTCG
LOC_Os08g33710-R	TCGTCGACGTTTTCCCGAAT
LOC_Os06g18790-F	CTCGCGAGCGAGAAGAAGG
LOC_Os06g18790-R	GAGCTGGTCGTTGCTCACG
LOC_Os02g47600-F	ACGCACACTTTGTGAGGACG
LOC_Os02g47600-R	CACGAGCTCGCAGGAAACAT
LOC_Os07g03710-F	TGTACTGTCAGCCGTATTTGCT
LOC_Os07g03710-R	CCATGCATGTAACCACGAAGGA
LOC_Os03g29240-F	GGGCGACTCATGGTCCTACA
LOC_Os03g29240-R	ACGTCTCCCTCGCACACATA
LOC_Os07g43670-F	TTCCAGGTGTACCAGTGCGT
LOC_Os07g43670-R	TAGAACGTGGGCAGCTTGAC
LOC_Os11g14950-F	TGTGAGGTTGCACGAACTGG
LOC_Os11g14950-R	AGGAAGGGAAAACCCACGGA
LOC_Os02g33850-F	GGGCATCACGTGCTGAAGAA
LOC_Os02g33850-R	ATCGGGGACACCTGGAACAT
LOC_Os07g36490-F	AGAAGCCGGTGAGGGCATAA
LOC_Os07g36490-R	AGTTGATGCTGCTGGTTGGG
LOC_Os10g11500-F	GCACCTTCCAAGGTCAGCCT
LOC_Os10g11500-R	GTAGTCCTGCGGCGTGTTCT
LOC_Os02g40710-F	GCGGCCAACTACATCTGCAA
LOC_Os02g40710-R	CACGAGGTAGGCCGAGAAGA
LOC_Os01g52010-F	TCGCGAAACTCCACCAAAGG
LOC_Os01g52010-R	CGTCAGGTGAGGCCAGTAGT
LOC_Os04g41970-F	CCCGTCATCATCGCCAAGTC
LOC_Os04g41970-R	TGATGCCGTTGTTCTTCGGG
LOC_Os01g16010-F	ACTGTCAACCGCGAGAGGAA
LOC_Os01g16010-R	TCTGACACAGCTCCTGCCAT
cpDNA-F	GTGTCTACGCGGTGGACTTG
cpDNA-R	ACAAAACGGTCCCTCCAACG
LOC_Os02g05880-F	CGGGTACATTGAGCTGCCTG
LOC_Os02g05880-R	CTTTCTTGACGCGGAGGCAT
LOC_Os03g40670-F	CGTCAAATGGTCAGACGGGA
LOC_Os03g40670-R	ATGAGTGACGATGGTGCGAA
LOC_Os03g05640-F	AGCTCTTCTTCGGATGGCTC
LOC_Os03g05640-R	TGGCAATCACGCTCTTGGG
LOC_Os10g30790-F	CTGCTCACCTACTACTGGCG

LOC_Os10g30790-R	ACCATCTGCTCCAGCTTGTC
LOC_Os12g44020-F	CTTCGATCACACCGTCCGTA
LOC_Os12g44020-R	ATGAGGCCGAACTTGAAGGG
LOC_Os01g47070-F	CCTGCAACTCCGGCCTATAC
LOC_Os01g47070-R	GAGGTTGAGGACAGGGGTCT
LOC_Os12g09620-F	GTGCTCCTGGTTGATTGGCT
LOC_Os12g09620-R	CAAACAAACAGCATCTGCATCC
LOC_Os05g33400-F	ACATGATGGAGGACAACCTGC
LOC_Os05g33400-R	TCGTTTGCCGGAACAGGAG
LOC_Os10g39300-F	TTCAGGAAGCAGGAGAGTGC
LOC_Os10g39300-R	TTCACCAACATGAGCAGCGT
LOC_Os01g25484-F	GACATCATCCCGGTGTGCAA
LOC_Os01g25484-R	AGCCTCCATTCCAAGTTCGT
LOC_Os03g52860-F	CAACCTTCTCTACGTGGTGGAC
LOC_Os03g52860-R	ATGAAGTTGTCGTCGAGGCTAT
LOC_Os05g15770-F	GGGGCATCATGGTCTGGAAC
LOC_Os05g15770-R	CTGATCCAGGAGACCAGGCT
PHR1-F	TCTGCAGAAGTACCGCACAG
PHR1-R	ATGCTCGCTTTCAGGTCCAA
PHR2-F	CAGCTGCGCAATCATCTGTC
PHR2-R	GAGGGCGAGGGTATAGCAAC
PHO1;1-F	AGCAATTTGAAGGGCAGCTTG
PHO1;1-R	ATCATGAGGTGTTGAGGTGGT
PHO1;2-F	TACCTTAAGCCACAGCAGCC
PHO1;2-R	TAGGCTGAGTTCCCAGTGGA
PHO1;3-F	ATGGCTTCAGACCGTCATCC
PHO1;3-R	GGTGCTCGTTTTCCAACCTG
NAT-PHO1;1-F	GCCCTTCAAATTGCTTCGAGAACT
NAT-PHO1;1-R	TCGCAACACCTGCAAAGATAGAGA
NAT-PHO1;3-F	GTGCCAACAGCAGAACAGATACAGT
NAT-PHO1;3-R	TTTTCAGCAATTTTCATGGTTACGG
NAT-PHO1;2-F	GGCTACTAGCTTGGTGCTCTTCTCC
NAT-PHO1;2-R	CAAGGCCACTATCTGTCCCTGTATG
OsActin 1-F	ACATCGCCCTGGACTATGACCA
OsActin 1-R	GTCGTACTCAGCCTTGGCAAT
